# Youth perception of air pollution and the evaluation of governmental environmental performance: Evidence from China

**DOI:** 10.1371/journal.pone.0345205

**Published:** 2026-04-21

**Authors:** Xiaoyan Li, Rongwang Guo

**Affiliations:** 1 School of Management and Engineering, Shanxi University of Finance and Economics, Taiyuan, China; 2 School of Agricultural Economics and Management, Shanxi Agricultural University, Jinzhong, China; Southwestern University of Finance and Economics, CHINA

## Abstract

Given that youth are central to societal development and environmental governance, their evaluation of governmental environmental performance is essential for policy refinement and enhanced efficacy. However, the mechanisms linking youth perception of air pollution to such evaluation remain underexplored. Drawing on data from the 2021 China Social Survey (CSS), this study employs an ordered logistic model to investigate this relationship. The results indicate that youth with a heightened perception of air pollution tend to give more negative evaluations of governmental environmental performance. Further mediation analysis using bootstrap methods identified three significant parallel mediators: residential satisfaction, environmental safety perception, and trust in local government. The relative contributions of these mediators were then decomposed and compared using the Karlson-Holm-Breen (KHB) method. These findings highlight the value of incorporating youth perspectives, advance the literature on public evaluations of environmental governance by delineating distinct psychological pathways, and provide empirical evidence for designing targeted policies.

## 1 Introduction

Environmental issues, with air pollution being a prominent example, constitute a pressing global challenge [1–3]. The detrimental effects of air pollution are extensive, severely impairing human health, diminishing quality of life, and causing significant damage to natural ecosystems. Reflecting its severity, the World Health Organization (2016) has identified air pollution as the single largest environmental health risk of this century [4]. Concurrently, young people are increasingly recognized as a crucial force in climate governance and an integral stakeholder in environmental policy processes [5]. Scholarly attention has correspondingly grown regarding the importance of youth engagement in environmental action. Characterized by considerable social activism and environmental awareness, this demographic plays a significant role in socio-economic development [6]. Their perceptions of air pollution not only inform personal lifestyle and behavioral choices but also hold substantial implications for broader environmental governance and policy improvement. Public evaluation of governmental environmental performance, defined as the subjective assessment of government policies and their implementation effectiveness in this domain, is a critical factor for future public administration [7]. Therefore, a rigorous investigation into how air pollution perceptions among youth influence their evaluations of governmental environmental performance is essential. This line of inquiry possesses considerable practical value for enhancing policy efficacy and advancing sustainable development.

Air pollution exerts wide-ranging adverse effects on the healthy development of young people, impacting their physical health, psychological well-being, cognitive performance, and social functioning. In terms of physical health, air pollution is a well-established risk factor for serious conditions such as cardiovascular disease [8], cancer [9], premature mortality [10], and mental health disorders including depression and anxiety [11]. It is also associated with lower levels of physical activity and increased sedentary behavior among adolescents [12]. Concerning mental health, evidence consistently links air pollution exposure to poorer psychological outcomes in youth, including reduced subjective well-being [13], higher rates of psychiatric symptoms, and sleep disturbances [14,15]. Socially, air pollution has been shown to inhibit prosocial behavior [16] and raise healthcare utilization and public medical expenditures [17]. Cognitively, air pollution exposure is associated with impaired cognitive function and lower academic achievement [18]. While existing research has extensively documented the direct health consequences of air pollution on young populations, it has largely overlooked the critical relationship between their subjective perception of pollution and their evaluation of governmental environmental action. Addressing this gap is essential for developing policies that effectively respond to both the ecological challenges and the societal perceptions that shape public accountability and policy support.

Existing research has examined the determinants of environmental governance performance from diverse perspectives. At the government level, strengthened anti-corruption efforts enhances the efficiency of local environmental governance [19], and reforms in government auditing systems significantly improve governance outcomes [20]. Furthermore, clearly defined policy objectives, active stakeholder participation, and effective policy tools contribute to improved environmental governance performance [21]. In terms of technology, increased internet usage may reduce residents’ satisfaction with environmental governance [7], whereas digital technologies significantly boost governance efficiency [22]. Regarding social participation and resource input, accumulating innovative human and capital resources markedly strengthens environmental governance performance [23]. Public engagement in environmental issues enhances pollution control effectiveness by raising governmental attention [24], and citizens’ awareness of environmental responsibility significantly improves their satisfaction with environmental governance [25]. Collectively, these studies suggest that governmental capacity, technological advancement, social participation, and policy instruments jointly shape environmental governance performance. However, little attention has been paid to the role of youth in influencing governmental environmental performance, particularly their satisfaction with environmental governance. Given the critical position of youth in socio-economic sustainable development, investigating their environmental governance satisfaction is essential for fostering their participation and optimizing governmental environmental performance.

Hence, despite extensive literature on environmental governance, the relationship between youth perception of air pollution and their evaluation of governmental environmental performance remains understudied within an integrated analytical framework. Although Wang et al. (2024) identified a negative association between public perception of air pollution and environmental governance evaluation under an ESG framework [26], their study did not focus specifically on youth populations, nor did it examine the underlying mechanisms at play. To address this gap, this research investigates the link between air pollution perception and governmental environmental performance evaluation among young people, with particular attention to the potential mediating roles of residential satisfaction, perceived environmental safety, and trust in government. Utilizing data from the 2021 Chinese Social Survey (CSS), the study analyzes the pathways through which youth perception of air pollution shapes their evaluation of governmental environmental performance. By doing so, it contributes to a deeper theoretical understanding of how young people perceive air pollution and judge the performance of environmental governance. The findings provide insights for encouraging youth participation and improving environmental governance effectiveness, thereby establishing a foundation for further research in this field.

This research contributes to the literature in two principal ways. First, it addresses a critical empirical gap by focusing on young people, a demographic whose distinct perceptions and evaluations of environmental governance have been underexplored. Synthesizing youth perceptions of air pollution with their evaluations of governmental environmental performance provides nuanced insights into the environmental cognition of this pivotal group. Second, it unpacks the mechanisms underlying this relationship. By identifying residential satisfaction, perceived environmental safety, and trust in local government as key mediators, the analysis clarifies how subjective experiences translate into institutional evaluations, thereby informing future theoretical development.

## 2 Materials and methods

### 2.1 Data and sample selection

The data for this study come from the 2021 Chinese Social Survey (CSS), a major longitudinal research project that has been conducted nationwide by the CASS Institute of Sociology since 2005. As a nationwide survey, the CSS systematically documents evolving trends in work, family life, and social attitudes among the Chinese public, thereby providing nuanced empirical data for social science research. Conducted biennially, the CSS utilizes a probability sampling approach for face-to-face household interviews, ensuring respondent anonymity throughout the process.

The CSS 2021 represents the eighth iteration of the survey, with a thematic focus on “Social Quality and Modernization”. It includes modules on social and political participation, which are relevant to the objectives of this study. The CSS 2021 covered 592 villages and communities across 30 provincial-level divisions in China (with the exception of Xinjiang), ultimately collecting 10,136 valid questionnaires. To align with the study’s focus on young people, the age range was set at 14–35 years, following the official definition of “youth” in China’s National Medium and Long-Term Youth Development Plan (2016–2025) [27], which is the first national-level youth development plan in China. Respondents older than 35 years or with missing values for key variables were excluded, yielding a final sample size of 1,269.

### 2.2 Ethics statement

The data used in this study were obtained from the 2021 Chinese Social Survey (CSS 2021), administered by the Institute of Sociology, Chinese Academy of Social Sciences (CASS). The design and implementation of CSS 2021 strictly adhered to the ethical guidelines established by the CASS Ethics Committee. All respondents provided written informed consent prior to participation. The survey was conducted on a voluntary basis, and respondents’ privacy, anonymity, and confidentiality were fully protected throughout the entire process.

The dataset employed in this research is a de-identified and encrypted public-use file distributed by the survey organizers. All directly identifying information has been removed, and the data are made available in accordance with open-access standards. As the present study involved only secondary analysis of these anonymized, publicly accessible data, no additional ethical approval or informed consent was required.

### 2.3 Measurement

#### 2.3.1 Dependent variable.

The variable was assessed by asking respondents to evaluate the statement: “Do you think the local government has done a good job in protecting the environment and controlling pollution?”. Youth responses were captured on a four-point scale ranging from 1 (“very poor”) to 4 (“very good”), with higher scores reflecting a more positive appraisal. This measurement approach has been validated and adopted by previous scholars [7,26].

#### 2.3.2 Independent variable.

Following Wang et al. (2024) and Fan (2024), youth perception of air pollution was gauged by the single item: “How serious do you consider air pollution to be in your current area?” [26,28]. Youth participants were recorded on a four-point scale ranging from 1 (“extremely serious”) to 4 (“not at all serious”). The variable was reverse-coded, so that higher scores indicate lower perceived air pollution severity.

#### 2.3.3 Mediating variables.

The study employs three mediating variables: residential satisfaction, environmental safety perception, and trust in local government, which are measured as follows.

Residential satisfaction, defined as residents’ overall contentment with their living environment, was operationalized by adapting the methodology of Omri et al. (2022) [29]. Using a single-item measure, respondents rated their satisfaction with local environmental conditions on a 10-point scale from 1 (“extremely dissatisfied”) to 10 (“extremely satisfied”), with higher scores reflecting greater satisfaction.

Environmental safety perception refers to an individual’s assessment of the safety level of their surrounding environment. It was measured using the question, “How do you rate the environmental safety in your current area?” Responses included four options: “very unsafe,” “slightly unsafe,” “slightly safe,” and “very safe,” which were coded from 1 to 4, respectively. Higher values indicate a higher perceived level of environmental safety.

Trust in local government constitutes a fundamental socio-political relationship, characterized by public expectations of governmental performance and the state’s corresponding responsiveness [30]. Within China’s fiscal decentralization framework, local governments assume primary responsibility for environmental governance, including pollution control and enforcement of central environmental standards [31]. To measure this construct, respondents answered the following question: “How much do you trust the following institutions (district or county government)?” Responses were recorded on a 4-point scale ranging from 1 (“completely distrust”) to 4 (“completely trust”), with higher values representing stronger trust.

#### 2.3.4 Control variables.

Drawing on previous research [31,32], this study selects several commonly used control variables in satisfaction analyses, including gender, age, marital status, education level, household registration status (hukou), political identity, socioeconomic status, and family size. Table 1 reports the variable definitions and descriptive statistics.

**Table 1:** Descriptive statistics of study variables (N = 1,269).

Variable	Variable Definition	Mean	SD	Min	Max
Evaluation of governmental environmental performance (gov_env)	1 = very poor; 2 = not so good; 3 = better; 4 = very good	2.931	0.662	1	4
Perception of air pollution (air_poll)	1 = extremely serious; 2 = somewhat serious; 3 = slightly serious; 4 = not at all serious	2.931	0.751	1	4
Residential satisfaction (res_env)	from 1 (extremely dissatisfied) to 10 (extremely satisfied)	7.379	1.831	1	10
Environmental safety perception (env_safety)	1 = very unsafe; 2 = slightly unsafe; 3 = slightly safe; 4 = very safe	3.035	0.573	1	4
Trust in local government (trust_gov)	1 = completely distrust; 2 = somewhat distrust; 3 = somewhat trust; 4 = completely trust	3.196	0.696	1	4
Gender	1 = male; 2 = female	1.586	0.493	1	2
Age	18-35	27.166	5.335	18	35
Marital status (married)	1 = unmarried; 2 = cohabitation or married; 3 = divorced or widowed	1.561	0.526	1	3
Educational level (edu)	1 = junior high school or below; 2 = senior high school; 3 = college degree or above	2.177	0.853	1	3
Hukou	1 = agricultural; 2 = non-agricultural; 3 = resident	1.587	0.801	1	3
Political identity (political)	1 = Communist Party of China member; 2 = Communist Youth League member; 3 = Democratic party member; 4 = Non-affiliate	3.206	1.112	1	4
Socioeconomic status (socio_status)	1 = upper; 2 = upper-middle; 3 = middle; 4 = lower-middle; 5 = lower	3.690	0.885	1	5
Family size (num)	1-17	4.487	1.805	1	17

## 3 Results

### 3.1 Baseline regression results

Given that the variables employed in this study are ordinal categorical variables, the ordered logistic regression model was utilized for the baseline analysis. Subsequently, the robustness of the results was examined by employing the ordered probit regression model and substituting dependent variable. These methods ensured that the findings were consistent and reliable across different model specifications.

The basic regression results displayed in Table 2 reveal that the independent variable, youth perception of air pollution (air_poll), exhibits a significantly positive coefficient in both Model 1 and Model 2. Specifically, the baseline estimate from Model 1 (without control variables) is 1.055 (p < 0.01), which confirms a statistically significant link between youth perception regarding air pollution and evaluation of governmental environmental performance (gov_env). When all control variables are included in Model 2, the coefficient slightly decreases to 1.038 (p < 0.01), yet it remains statistically significant. This analysis demonstrates that an increase in youth perception of air pollution is negatively associated with their approval of governmental environmental performance.

**Table 2:** Basic regression results.

	gov_env	
	(1)	(2)
air_poll	1.055***	1.038***
	(0.087)	(0.088)
gender		−0.085
		(0.126)
age		−0.031*
		(0.016)
married		0.198
		(0.169)
edu		−0.061
		(0.087)
hukou		0.070
		(0.348)
political		−0.099*
		(0.064)
socio_status		−0.091
		(0.184)
num		−0.035
		(0.037)
Province effect	YES	YES
N	1,269	1,269
R^2^	0.087	0.091

Note: standard errors are in the parentheses, * p < 0.10; ** p < 0.05; *** p < 0.01.

To ensure the robustness of the findings, two approaches were employed. First, the ordered probit model was estimated as a robustness check to complement the ordered logistic regression results. Second, the dependent variable was replaced with a measure of the public’s satisfaction with the government to further assess the robustness of the results. Specifically, the item “Overall, how would you rate the performance of the local government?” was used, with responses ranging from 1 (very poor) to 4 (very good), where higher values indicate higher levels of satisfaction.

The results are presented in Table 3. Models 1 and 2 display the regression outcomes using the ordered probit model, while Models 3 and 4 present the results with the dependent variable replaced by the public’s satisfaction with the government. As shown, the coefficient of youth perception of air pollution remains significant at the 1% level across all models. These consistent findings confirm the robustness of the initial model results and provide a solid foundation for further research.

**Table 3:** Robustness test of basic regression results.

	Ordered probit model		Alternative gov_env	
	(1)	(2)	(3)	(4)
air_poll	0.555***	0.548***	0.705***	0.682***
	(0.047)	(0.047)	(0.089)	(0.090)
gender		−0.043		−0.074
		(0.070)		(0.134)
age		−0.017*		−0.036**
		(0.009)		(0.017)
married		0.115		0.117
		(0.094)		(0.181)
edu		−0.030		−0.226
		(0.049)		(0.094)
hukou		0.045		0.185
		(0.042)		(0.080)
political		−0.054		−0.119
		(0.036)		(0.068)
socio_status		−0.047		−0.217
		(0.038)		(0.073)
num		−0.020		−0.028
		(0.021)		(0.040)
Province effect	YES	YES	YES	YES
N	1,269	1,269	1,269	1,269
R^2^	0.084	0.089	0.054	0.066

Note: standard errors are in the parentheses, * p < 0.10; ** p < 0.05; *** p < 0.01.

### 3.2 Mediation analysis

The mediation analysis was conducted using the bootstrap resampling method to assess the indirect and direct effects. Specifically, the indirect effect was evaluated through the product of coefficients approach, and the significance of the mediation effect was determined by constructing a 95% confidence interval based on 1,000 bootstrap replications. This approach provides a robust estimation of the mediation effect and ensures the reliability of the results.

#### 3.2.1 The mediating role of residential satisfaction.

To investigate the underlying mechanism, this study first examined the mediating role of residential satisfaction between youth perception of air pollution and their evaluation of governmental environmental performance. As shown in Table 4, bootstrapping analysis with 1,000 resamples revealed a significant indirect effect of air pollution perception on the evaluation of governmental environmental performance through residential satisfaction (indirect effect = 0.138, SE = 0.017, 95% CI [0.105, 0.170], z = 8.249, p < 0.01). The direct effect of air pollution perception on performance evaluation, after controlling for the mediator, also remained significant (direct effect = 0.167, SE = 0.029, 95% CI [0.109, 0.224], z = 5.690, p < 0.01). The significance of both the indirect and direct effects indicates that residential satisfaction functions as a partial mediator among the youth. This suggests that youth perception of air pollution not only directly lowers their evaluation of the governmental environmental performance but also exerts a negative influence indirectly by impairing their residential satisfaction.

**Table 4:** The mediating effect of residential satisfaction.

Effect Type	Unstandardized Coefficient	SE	Z-value	P > |Z|	95% CI
Indirect effect	0.138	0.017	8.249	<0.01	[0.105, 0.170]
Direct effect	0.167	0.029	5.690	<0.01	[0.109, 0.224]

Note: N = 1,269; bootstrap replications = 1000; CI = confidence interval.

#### 3.2.2 The mediating role of environmental safety perception.

This study further examined the mediating role of environmental safety perception. As shown in Table 5, bootstrapping analysis with 1,000 replications revealed a significant indirect effect (indirect effect = 0.098, SE = 0.014, 95% CI [0.070, 0.126], z = 6.908, p < 0.01). The direct effect was also significant and notably larger in magnitude (direct effect = 0.206, SE = 0.027, 95% CI [0.152, 0.259], z = 7.541, p < 0.01). The statistical significance of both effects confirms that environmental safety perception functions as a partial mediator. These results indicate two concurrent pathways through which youth perception of air pollution influences their evaluation of governmental environmental performance, including a direct pathway and an indirect pathway operating through reduced environmental safety perception.

**Table 5:** The mediating effect of environmental safety perception.

Effect Type	Unstandardized Coefficient	SE	Z-value	P > |Z|	95% CI
Indirect effect	0.098	0.014	6.908	<0.01	[0.070, 0.126]
Direct effect	0.206	0.027	7.541	<0.01	[0.152, 0.259]

Note: N = 1,269; bootstrap replications = 1000; CI = confidence interval.

#### 3.2.3 The mediating role of trust in local government.

The analysis extended to investigate local government trust as a mediator. As shown in Table 6, results from bootstrapping (1,000 replications) confirmed a significant indirect pathway (indirect effect = 0.041, SE = 0.008, 95% CI [0.024, 0.057], z = 4.472, p < 0.01). Notably, the direct effect of air pollution perception on evaluation of governmental environmental performance remained strong and significant (direct effect = 0.263, SE = 0.028, 95% CI [0.209, 0.318], z = 9.117, p < 0.01). This evidence supports a model of partial mediation through local government trust. The findings suggest that for young people, the perception of air pollution damages their trust in local government, which in turn contributes to a more negative evaluation of environmental performance. However, the substantial direct effect indicates that this loss of trust is not the sole explanation, as perceptions of pollution also exert a significant direct influence on their evaluations.

**Table 6:** The mediating effect of trust in local government.

Effect Type	Unstandardized Coefficient	SE	Z-value	P > |Z|	95% CI
Indirect effect	0.041	0.008	4.472	<0.01	[0.024, 0.057]
Direct effect	0.263	0.028	9.117	<0.01	[0.209, 0.318]

Note: N = 1,269; bootstrap replications = 1000; CI = confidence interval.

#### 3.2.4 Decomposition of multiple mediating effects (KHB method).

The above mediation analysis indicated that residential satisfaction, environmental safety perception, and trust in local government each serve as significant mediators in the relationship between youth perception of air pollution and governmental environmental performance. To quantify the mediation effects and the relative contributions of each mediator, the Karlson-Holm-Breen (KHB) method was employed. This method is particularly suitable for effect decomposition in nonlinear models [33].

As shown in Table 7, the KHB decomposition reveals that the total effect of youth perception of air pollution on governmental environmental performance (coefficient = 1.153, p < 0.01) is largely mediated through these parallel pathways. Specifically, 61.32% of the total effect operates indirectly through the mediators. Among them, residential satisfaction constitutes the most substantial channel, accounting for 48.37% of the total indirect effect. Environmental safety perception contributes 38.90% of the indirect effect, while trust in local government explains a smaller yet significant portion (12.73%).

**Table 7:** Decomposition of total effects (KHB).

Effects		Coefficient	Percentage of Indirect Effect	Percentage of Total Effect	Percentage of Indirect Effect
Reduced (Total effect)		1.153*** (0.088)		100%	
Full (Direct effect)		0.446*** (0.094)		38.68%	
Difference (Indirect effect)		0.707*** (0.065)	100%	61.32%	100%
	Residential satisfaction	0.342*** (0.050)	48.37%		48.37%
	Environmental safety perception	0.275*** (0.038)	38.90%		38.90%
	Trust in local government	0.090*** (0.021)	12.73%		12.73%

Note: standard errors are in the parentheses, * p < 0.10; ** p < 0.05; *** p < 0.01.

These findings suggest that the relationship between youth perception of air pollution and governmental environmental performance is primarily transmitted through perceptual and evaluative mechanisms. Residential satisfaction and environmental safety perception play dominant roles in this mediation process, highlighting the importance of subjective evaluations of living conditions and environmental quality. Meanwhile, trust in local government, despite its smaller share, is a significant mediator that plays an important yet less dominant role in shaping youth perception of environmental performance.

## 4 Discussion

Drawing on data from the CSS2021, this study investigates the impact of youth perception of air pollution on their evaluation of governmental environmental performance. The results reveal that a heightened youth perception of air pollution severity significantly undermines their evaluation of governmental environmental performance, demonstrating a clear adverse relationship. Mediation analyses identify three significant pathways, namely residential environmental satisfaction, environmental safety perception, and trust in local government, through which air pollution perception among youth shapes their evaluation of governmental environmental performance. These findings offer crucial insights into the multifaceted nature of public evaluations of environmental governance by elucidating these mechanisms.

First, this study demonstrates that youth perception of air pollution significantly undermines their evaluation of governmental environmental performance, thereby extending to a younger population a relationship previously documented in the general public by Yao et al. (2022), Zhu et al. (2023), and Zhang et al. (2024) [26,32,34]. This finding resonates with the national prioritization of ecological governance, as outlined in the Beautiful China Youth Action Plan (2024–2028) [35]. Specifically, it empirically highlights youth evaluation of governmental environmental performance as a key area for enhancing public engagement. Consequently, the results collectively affirm that incorporating youth perspectives into environmental policymaking, particularly in air pollution control, is critical for building governance systems responsive to this demographic’s concerns.

Secondly, this study reveals that residential environmental satisfaction serves as a significant mediator through which air pollution perception among youth influences their evaluation of governmental environmental performance. This finding extends the literature by moving beyond the well-documented direct effects of residential satisfaction on psychological well-being, such as life satisfaction and happiness [36,37]. It reveals a more complex mediating mechanism, positioning residential satisfaction as a pivotal bridge that translates youth perception of air pollution into their evaluation of environmental governance. This mechanism finds its macro-level corroboration in the finding that air pollution directly drives population outflow [38], a phenomenon which can be interpreted as a behavioral manifestation of critically low residential satisfaction. Collectively, this suggests that the quality of the living environment not only affects personal well-being but also shapes evaluations of governmental performance, with significant societal consequences.

Thirdly, this analysis identifies environmental safety perception as a critical mechanism through which youth perception of air pollution influences their evaluation of governmental environmental performance, linking personal risk assessment to political evaluations. The importance of environmental safety for young people’s psychological well-being and long-term development is well-established in the literature. Previous research has demonstrated that perceptions of unsafe community environments can hinder adolescent development [39], while other studies have examined how individual perceptions of environmental safety affect mental health outcomes [40]. However, few studies have connected environmental safety perception specifically to the evaluation of environmental governance. Building on youths’ heightened sensitivity to both air pollution and environmental governance, this study verifies the mediating role of environmental safety perception between youth perception of air pollution and their evaluation of governmental environmental performance. These findings highlight environmental safety perception as a key subjective cognitive factor that not only influences daily behaviors and attitudes among youth but also establishes an important connection between their perception of environmental issues and evaluation of governmental environmental performance.

Moreover, trust in local government mediates the relationship between youth perception of air pollution and their evaluation of governmental environmental performance. This finding corroborates existing literature on the fundamental role of political trust in environmental governance. Previous studies indicate that air pollution leads Chinese citizens to perceive inadequate governmental protection of their basic needs and well-being, thereby eroding trust in local authorities [25,32,41]. Conversely, strong political trust can enhance governmental motivation to implement effective measures while reducing policy enforcement resistance [42]. Unlike Wang et al. (2024), who examined the differential moderating effects of trust across government levels, this research focuses specifically on the mediating role of trust in local government [26]. This focus is theoretically justified as local government and young people serve as primary policy implementers and bear direct responsibility for environmental governance. Substantial evidence confirms that public trust in local government is essential for fostering civic participation and improving governance effectiveness [31,32,43]. The current analysis thus extends this research tradition by not only empirically establishing trust in local government as a key mechanism, but also by focusing on young people to demonstrate how environmental perceptions are translated into political evaluations through this channel. In practical terms, these findings underscore that strengthening youth trust requires local government to not only demonstrate environmental commitment through transparent and effective actions but also to balance developmental goals with sustainable governance, thereby enhancing both public confidence and policy efficacy.

Lastly, it is noteworthy that the strengths of these parallel pathways differed substantially. Residential satisfaction emerged as the predominant mediator, followed by environmental safety perception, whereas trust in local government played a markedly weaker role. This hierarchy can be interpreted through a framework of psychological proximity and the multidimensional nature of political constructs. Specifically, the dominance of residential satisfaction and environmental safety perception suggests that for youth, environmental concerns are translated most effectively into governance evaluations through the direct, personal lens of lived experience. By contrast, the more limited role of trust in local government likely stems from its nature as a broader, aggregate attitude that is less sensitive to fluctuations in any single policy domain. Thus, the psychological process linking pollution perception among youth to evaluation of governmental environmental performance is not uniformly distributed but is disproportionately channeled through pathways of greatest personal salience.

## 5 Conclusion and policy implications

### 5.1 Conclusion

Based on data from the CSS2021, this study employs an ordered logistic regression model to examine the association between youth perception of air pollution and their evaluation of governmental environmental performance. The findings reveal that higher levels of perceived air pollution are associated with significantly lower evaluations of governmental environmental performance. To unpack the mechanisms behind this association, a series of mediation analyses were conducted. The significance of the parallel mediation pathways was tested using the Bootstrap method, confirming that residential satisfaction, environmental safety perception, and trust in local government all serve as significant mediators. Furthermore, the Karlson-Holm-Breen (KHB) decomposition method was applied to quantify and compare the relative contributions of each mediator. This step allows for a precise decomposition of the total effect into its direct and indirect components. Collectively, these mechanisms illustrate how environmental perception is translated into political evaluation among youth.

Despite these contributions, several limitations should be acknowledged. First, the cross-sectional nature of the CSS2021 data constrains causal inference and precludes the examination of temporal dynamics. Although the robustness checks confirm the observed associations, they cannot fully address endogeneity concerns, such as omitted variable bias or reverse causality. Therefore, the findings are best interpreted as robust correlations, with the proposed causal mechanisms remaining a testable hypothesis for future longitudinal research.

Second, while three parallel mediators were identified, their potential chain or sequential relationships remain unexamined. Theoretically, residential satisfaction, environmental safety perception, and trust in local government may be interrelated. Future research should therefore model their potential interdependencies, such as whether environmental concerns reduce satisfaction which in turn affects trust, to build a more integrated theoretical model linking youths perception of air pollution to their evaluation of governmental environmental performance.

Thirdly, the measures of youth perception of air pollution and their evaluation of governmental environmental performance rely on self-reported survey data. While such perceptual measures effectively capture subjective evaluations, they may be influenced by variations in individual environmental awareness, knowledge levels, and personal experiences. Future research could incorporate objective measures of air quality and environmental governance performance to provide a more robust assessment of the relationship between air pollution perception and governmental evaluation.

### 5.2 Policy implications

The findings underscore the necessity of addressing environmental concerns among youth to improve their evaluation of environmental governance. Given young people’s crucial role in shaping future societal development, policymakers should prioritize their inclusion in environmental policy processes through structured participatory platforms and targeted educational programs. Specific measures include establishing youth advisory bodies, organizing regular public consultations, and utilizing digital communication channels to effectively solicit and incorporate their feedback. Recognizing youth as essential stakeholders in sustainable development helps cultivate their sense of collective responsibility and engagement.

Therefore, sustained efforts to improve air quality remain imperative. Policy measures should prioritize stricter controls on industrial and vehicular emissions, accelerate the transition to clean energy, and enhance the accessibility and quality of public transportation. These interventions not only directly mitigate air pollution but also contribute to creating healthier and more livable residential environments for young people.

Furthermore, the identified mediating pathways suggest several targeted approaches. Enhancing residential environmental satisfaction among youth requires urban planning that prioritizes green spaces, noise reduction, and improved sanitation infrastructure. Strengthening environmental safety perception among youth necessitates comprehensive monitoring systems with transparent public reporting, complemented by educational campaigns demonstrating concrete governmental actions. Building youth trust in local government demands consistent policy implementation, accessible grievance mechanisms, and visible accountability measures.

These integrated approaches addressing both objective environmental conditions and subjective perceptions can significantly improve youth evaluation of governmental environmental performance. By simultaneously tackling air quality concerns while reinforcing the mediating factors of residential satisfaction, safety perception, and institutional trust among youth, policymakers can establish a virtuous cycle of environmental improvement and young people’s confidence in governance. This, in turn, creates a stronger foundation for sustainable development.

## Supporting information

S1 File Data. (ZIP)
